# Antibacterial and Anticorrosive Hydrogel Coating Based on Complementary Functions of Sodium Alginate and g-C_3_N_4_

**DOI:** 10.3390/molecules29174192

**Published:** 2024-09-04

**Authors:** Zishuai Hu, Baochen Han, Jianhui Li, Dan Liu, Jian Qi

**Affiliations:** 1 Hebei Short Process Steelmaking Technology Innovation Center, School of Materials Science and Engineering, Hebei University of Science and Technology, Shijiazhuang 050018, China; hzis1999@163.com (Z.H.); lijianhui_97@163.com (J.L.); 2 State Key Laboratory of Biochemical Engineering, Institute of Process Engineering, Chinese Academy of Sciences, Beijing 100049, China

**Keywords:** hydrogel coating, g-C_3_N_4_, photocatalysis, antibacterial, anticorrosive

## Abstract

Graphitic carbon nitride (g-C_3_N_4_, CN) has emerged as a promising photocatalytic material due to its inherent stability, antibacterial properties, and eco-friendliness. However, its tendency to aggregate and limited dispersion hinder its efficacy in practical antibacterial applications. To address these limitations, this study focuses on developing a composite hydrogel coating, in which sodium alginate (SA) molecules interact electrostatically and through hydrogen bonding to anchor CN, thereby significantly improving its dispersion. The optimal CN loading of 35% results in a hydrogel with a tensile strength of 120 MPa and an antibacterial rate of 99.87% within 6 h. The enhanced mechanical properties are attributed to hydrogen bonding between the -NH2 groups of CN and the -OH groups of SA, while the -OH groups of SA facilitate the attraction of photogenerated holes from CN, promoting carrier transfer and separation, thereby strengthening the antibacterial action. Moreover, the hydrogel coating exhibits excellent antibacterial and corrosion resistance capabilities against Pseudomonas aeruginosa on 316L stainless steel (316L SS), laying the foundation for advanced antimicrobial and anticorrosion hydrogel systems.

## 1. Introduction

Microbiologically influenced corrosion (MIC) represents a significant form of failure in metallic materials, potentially leading to substantial economic losses [1]. This type of corrosion is particularly prevalent in marine environments, posing a considerable threat to offshore installations and structures [1,2,3]. In contrast to the traditional protective coatings, photocatalytic coatings offer a highly efficient and environmentally friendly alternative, as they harness solar energy to facilitate antibacterial and anticorrosive processes. Consequently, they are a promising option for combating microbial corrosion [4,5,6].

Graphitic carbon nitride (g-C_3_N_4_, CN) is a semiconductor material distinguished by excellent photoelectric properties as well as high thermal and chemical stability [7]. However, the application of CN is constrained by several defects, including its tendency to stack, a high recombination rate of photogenerated carriers, and the inability to form films [8]. Sodium alginate (SA) is a natural polymer frequently employed as a building block for hydrogels [9,10]. CN-SA hydrogel combines the advantages of both materials, resulting in a composite hydrogel that provides a stable platform for CN loading. In applications such as photocatalytic water splitting for hydrogen production and the degradation of organic pollutants, this composite material can offer an enhanced photocatalytic performance [11]. Additionally, it can improve the photocatalytic activity of CN, while increasing the adsorption capacity for microorganisms due to its porous structure. This enhancement is particularly significant for the design of water treatment systems and bioreactors [12]. In sewage treatment, this composite material can serve as an effective microbial carrier and improve the removal efficiency of pollutants [13]. However, the loading amount of CN significantly affects the properties of the hydrogel, particularly its mechanical properties, which are closely related to the protective effect of the coating. Nevertheless, there are currently few studies that focus on the impact of CN loading on hydrogel systems, especially SA hydrogels, indicating the pressing need for relevant research.

In an additional phase, carbon nitride particles can be integrated into the sodium alginate hydrogel network through either physical adsorption or chemical bonding [14]. Physical adsorption encompasses non-covalent bonding interactions, such as van der Waals forces and electrostatic interactions, whereas chemical bonding involves covalent bonds formed between the carboxyl groups on the sodium alginate molecular chain and the functional groups on the carbon nitride surface [15]. Other researchers [16,17] have tried to introduce two-dimensional nanomaterials into SA to prepare composite membranes with a good performance and strong stability. Ye et al. [18] developed carbon nanotubes/phenolic resin spheres through carbonization and activation, exhibiting robust mechanical strength, a medium-to-high pore volume, and the efficient adsorption of medium-molecular-weight toxins. Similarly, Jiang et al. [19] crafted a GO/SA/PAM hydrogel with excellent elasticity, capable of adsorbing heavy metal ions. These two methods not only enhance the mechanical strength of the hydrogel, but also improve its durability against specific environmental stimuli.

Photocatalytic antibacterial activity is the primary reason that carbon nitride hydrogels exhibit antibacterial and corrosion-resistant effects [20]. This photocatalytic antibacterial effect primarily relies on carbon nitride’s ability to generate electron–hole pairs under illumination, which subsequently participate in the photocatalytic reaction [21]. The photocatalytic performance is closely related to the system’s light absorption range, charge transfer efficiency, and the number and distribution of surface reaction active sites. Huang et al. [22] modified the amination surface of CN by treating it with monoethanolamine, which increased the number of reactive sites and significantly enhanced the separation efficiency of the photogenerated carriers. Li et al. [23] obtained the surface-alkalized CN by introducing KCl and NH_4_Cl during the thermal condensation polymerization of melamine. The hydroxyl groups grafted on the surface of CN can capture the photogenerated holes, further inhibiting the recombination of photogenerated carriers. The distribution and effective surface area of CN within a hydrogel system are closely related to the loading amount of CN, which directly influences the photocatalytic antibacterial efficacy of the hydrogel system [24]. Therefore, a comprehensive investigation into the relationship and mechanisms influencing the loading of CN and the photocatalytic performance of hydrogel systems is of great significance for the development of new, high-efficiency photocatalytic antibacterial coatings.

This study aims to develop an eco-friendly composite hydrogel coating utilizing SA and CN, while thoroughly investigating the interaction mechanisms between CN and SA. Additionally, we examined the effects of varying loading amounts on the mechanical properties and photocatalytic antibacterial performance of the composite hydrogel. Through these investigations, we aspire to provide a theoretical foundation for the advancement of new, efficient, and high-performance antibacterial hydrogels, thereby broadening their applications in the field of MIC.

## 2. Results

### 2.1. Form and Structure

**Figure 1:**
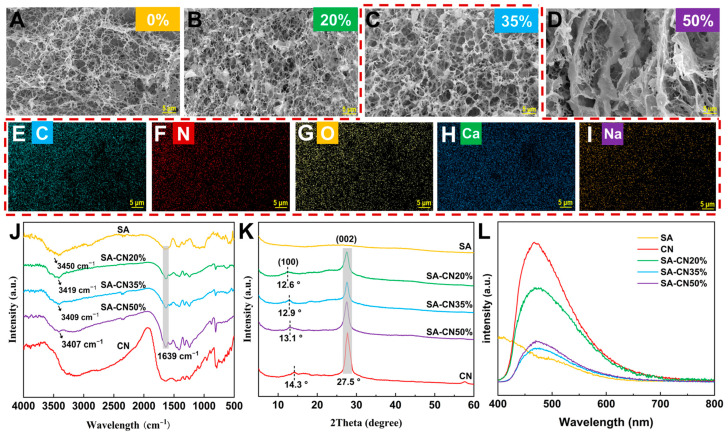
SEM images of SA (**A**), SA-CN20% (**B**), SA-CN35% (**C**), and SA-CN50% (**D**) are presented, along with an EDS image of SA-CN35% (**E**–**I**). Additionally, the FT-IR spectrum (**J**), the XRD data (**K**), and the PL spectrum (**L**).

The morphologies of SA, SA-CN20%, SA-CN35%, SA-CN50%, and CN were observed using scanning electron microscopy (SEM), as illustrated in Figure 1A–D. Figure 1A reveals that SA exhibits a distinctive three-dimensional network structure, characterized by a finely cross-linked network with numerous gaps. This structure results from the gradual acidification of CaCO_3_ and the cross-linking of Ca^2+^ with SA [25]. Additionally, the presence of substantial amounts of CO_2_ (Figure 1B) illustrates that CN is well dispersed within the SA-Ca^2+^ network structure, confirming the successful incorporation of CN into the SA hydrogel. When comparing SA-CN20% to SA-CN35%, it is evident that the hydrogel grid cross-linked lines formed by SA-CN35%, which contains a CN loading of 35% (Figure 1C), exhibit greater strength. This enhancement in strength can be attributed to CN serving as an additional phase that increases the overall CN loading and provides more cross-linking sites for SA during the cross-linking process, thereby resulting in a more robust mesh structure [26]. However, when the loading amount of CN reaches 50% (Figure 1D), the SA-CN50% hydrogel grid exhibits the stacking of excess CN, which hinders the cross-linking of the hydrogel and results in a scattered grid formation. To further confirm the elemental composition, the results of energy-dispersive X-ray spectroscopy (EDS) (Figure 1E–I) indicate that C, O, N, Na, and Ca are evenly distributed in the SA-CN35% composite hydrogel, as shown in Figure S1. This demonstrates that the Ca^2+^-CN composite material was successfully prepared.

The functional groups and complex chemical structure of the hydrogel were analyzed using Fourier-transform infrared spectroscopy (FT-IR), as shown in Figure 1J. For SA, the peak at 3450 cm^–1^ corresponds to the OH stretching vibration, the peak at 2940 cm^–1^ is associated with the C–H stretching vibration, and the peak at 1639 cm^–1^ corresponds to the O=C stretching vibration in the carboxyl group [27]. In the case of CN, the broad peak observed between 3000 and 3550 cm^−1^ is attributed to the stretching vibration of the NH bond in the terminal amino group. Additionally, the series of characteristic peaks ranging from 1700 to 1200 cm^−1^ corresponds to the C–N stretching vibrations present in the heterocyclic structure [28]. For the SA-CN20%, SA-CN35%, and SA-CN50% samples, the series of characteristic peaks at 1639 cm^–1^ and in the range of 1700 to 1200 cm^–1^ are evident in all the SA-CN hydrogels, indicating the formation of a composite material between SA and CN. Notably, new characteristic peaks appear at 3419 cm^–1^, 3409 cm^–1^, and 3407 cm^–1^, with the peak positions exhibiting a certain degree of shift compared to those observed before recombination. This shift can be attributed to the interaction between the -NH_2_ group at the CN end and SA. The formation of hydrogen bonds between the -OH groups facilitates the stable anchoring of CN within the SA matrix, thereby enhancing the stability of the network structure and promoting the more uniform distribution of CN [29,30,31]. In comparison to SA-CN20% and SA-CN35%, the 3000–3550 cm^–1^ region of SA-CN50% becomes more pronounced. This enhancement may be attributed to the excessive loading of CN, which leads to the aggregation of CN and results in the terminal amino groups (NH) becoming more dominant, thereby increasing the peak value [32,33].

The crystal structure of the material was analyzed using X-ray diffraction (XRD), as illustrated in Figure 1K. The XRD pattern of SA exhibits only broad lines between 5° and 60°, indicating its amorphous nature [34]. In contrast, the diffraction pattern of CN displays two distinct peaks at 14.3° (corresponding to the 100 crystal plane) and 27.5° (corresponding to the 002 crystal plane), which confirms the successful preparation of CN [35]. The peaks of SA-CN20%, SA-CN35%, and SA-CN50% exhibit weakening at 27.5°. This phenomenon is attributed to the hydrogen bonding between the -OH groups in the SA molecule and the -NH_2_ groups at the CN end. Such interactions occur during the cross-linking of SA, which promotes the dispersion of CN, anchors the CN within the SA matrix, enhances the mechanical properties of the coating, and significantly addresses the interlayer stacking issue of CN [36,37]. The peak at 27.5° of SA-CN50% is slightly enhanced, suggesting that excessive CN loading has been accumulated, which aligns with the findings from FT-IR and SEM analyses. Concurrently, the peak value at 14.3° appears to be shifted, potentially due to physical effects that facilitate the adsorption of SA onto the CN surface. This alteration may change the crystal orientation of CN or induce inhomogeneity in the grain size, resulting in a shift to a lower 2θ value [38].

The carrier separation of the material was investigated using photoluminescence spectroscopy (PL), as shown in Figure 1L. SA has no characteristic peaks; SA-CN20%, SA-CN35%, and SA-CN50% all have peaks near 475 nm, indicating the successful loading of CN onto SA. Generally, a lower photoluminescence (PL) intensity suggests that the recombination efficiency of the photoexcited electrons and holes is relatively low, indicating enhanced photocatalytic activity [39]. The peak intensity of the SA-CN samples is lower than that of CN. This can be attributed to the presence of a significant amount of -OH groups in SA, which attract the photogenerated holes produced by CN and improve the carrier separation efficiency [40]. Among the samples, SA-CN35% exhibits the lowest spectral intensity, which suggesting it possesses the highest photocatalytic activity. In contrast, the peak intensity of SA-CN50% increases due to the excess of CN, which leads to stacking, and subsequently impedes carrier separation.

### 2.2. Light Absorption and Optoelectronic Properties

**Figure 2:**
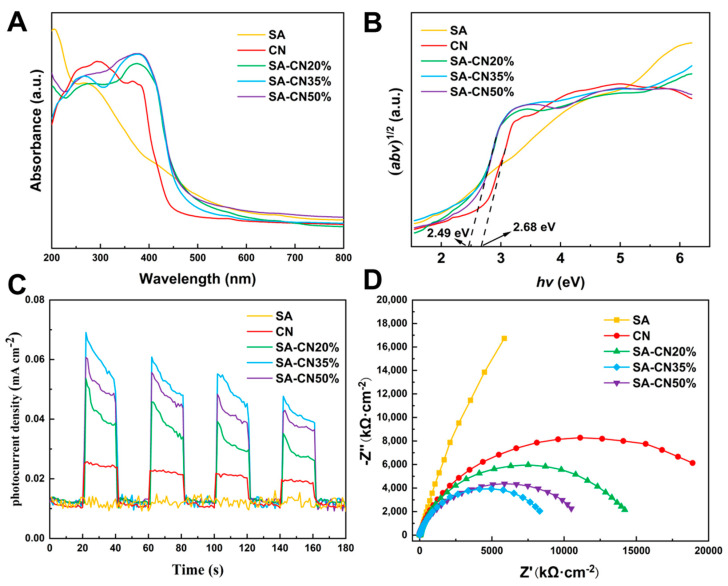
The UV-visible diffuse reflectance spectra (**A**) of SA, SA-CN20%, SA-CN35%, and SA-CN50% are presented, along with the corresponding band gap energy (**B**), the TPC (**C**), and EIS (**D**).

The light absorption properties of the material were investigated using ultraviolet-visible diffuse reflectance spectroscopy (UV-Vis DRS), as illustrated in Figure 2A. CN powder exhibits strong absorption in the visible light region, with its absorption band edge located at approximately 465 nm. In comparison to CN, the absorption wavelength of SA-CN was red-shifted to 493 nm, resulting in a significant enhancement of absorption in the mid- and long-wavelength visible light ranges. This improvement can be attributed to the three-dimensional network structure of SA, which enhances the layer-stacking structure of CN, thereby increasing the conductivity and photocatalytic activity of the material [41]. The band gap energy (Eg) of the CN and SA-CN materials can be determined using the Kubelka–Munk function, as illustrated in Figure 2B. The band gap energy values for SA-CN20%, SA-CN35%, and SA-CN50% are all 2.49 eV, which is lower than that of CN at 2.68 eV. This indicates that SA-CN exhibits enhanced light absorption performance under visible light irradiation.

Under light irradiation, the photocatalyst generates a transient photocurrent (TPC), which serves as reliable evidence for evaluating the separation and transfer of photogenerated charge carriers [42], as illustrated in Figure 2C. The samples CN, SA-CN20%, SA-CN35%, and SA-CN50% all produce a distinct photocurrent upon illumination. In contrast, because SA is not a semiconductor, it does not exhibit a photocurrent response. As the loading of CN increases, the photocurrent exhibits a significant increase. The average photocurrent of SA-CN35% is approximately three times greater than that of CN. This observation indicates that a greater number of photogenerated carriers are produced, further supporting the notion that the abundant hydroxyl groups on the surface of SA can effectively attract the photogenerated holes generated by CN. Consequently, this interaction leads to the generation of more photogenerated electrons and enhances the separation efficiency of the electrons (e^-^) and holes (h^+^). This results in enhanced photocatalytic activity [43]. However, when the loading of CN reaches 50%, the photocurrent intensity decreases compared to that of SA-CN35%. This decline is attributed to excessive CN loading, which negatively impacts its dispersion and leads to CN stacking, thus affecting the structural integrity of SA-CN. The conductivity of CN diminishes, which subsequently reduces the efficiency of photoelectric separation and leads to a decrease in the photocurrent intensity.

The optoelectronic properties of the material were further investigated using electrochemical impedance spectroscopy (EIS) [44]. As illustrated in Figure 2D, CN exhibits the largest impedance arc radius, while SA-CN20% follows, and SA-CN35% displays the smallest impedance arc radius. The radius of the semicircle represents the charge transfer resistance, indicating that SA-CN35% demonstrates higher efficiency in photogenerated electron transfer. Consequently, mobility is enhanced, leading to a significant improvement in photocatalytic activity. The electrochemical impedance of SA-CN50% falls between that of SA-CN20% and SA-CN35%. This phenomenon may be attributed to the excessive addition of CN, which leads to interlayer stacking, and subsequently increases the charge transfer resistance.

### 2.3. Mechanical and Swelling Properties

**Figure 3:**
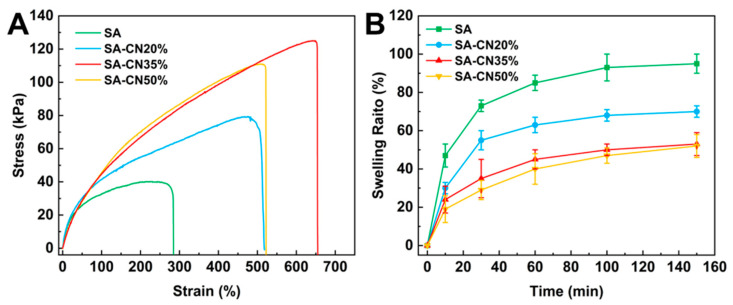
The tensile strength (**A**) of SA, SA-CN20%, SA-CN35%, and SA-CN50% was analyzed, alongside their swelling performance (**B**).

The mechanical properties of hydrogel coatings directly influence their stability in water [45]. As illustrated in Figure 3A, the tensile stress of the SA hydrogel was measured at 40 kPa, with a strain of 283%. Notably, when the concentration of CN reaches 35%, the tensile strength increases significantly, achieving a value of 120 kPa, which is three times greater than that of the SA hydrogel. This phenomenon can be attributed to the incorporation of g-C_3_N_4_ as a nano-reinforcement agent in the system. The amino groups at the CN terminal enhance the formation of hydrogen bonds with the hydroxyl groups of SA, thereby increasing the structural stability and improving the mechanical properties of the composite hydrogel. However, the tensile strength of SA-CN50% decreased, indicating that the excessive loading of CN led to CN stacking, which inhibited the formation of hydrogen bonds, and ultimately resulted in a reduction in the tensile strength of the coating. This observation is consistent with the findings from FT-IR and XRD analyses.

Additionally, less swelling is a critical requirement for the preparation of coatings [46]. As illustrated in Figure 3B, SA achieved swelling equilibrium, with a swelling rate of 95% after 150 min of the swelling test, and it nearly completely recovered. Following the swelling test, the swelling rates of SA-CN20%, SA-CN35%, and SA-CN50% significantly decreased to 40%. The hydrogen bonds formed between CN and SA upon loading contribute to a tighter internal structure of the hydrogel, enhancing the strength of the grid and complicating recovery after water loss.

### 2.4. Photocatalytic Antibacterial Performance

**Figure 4:**
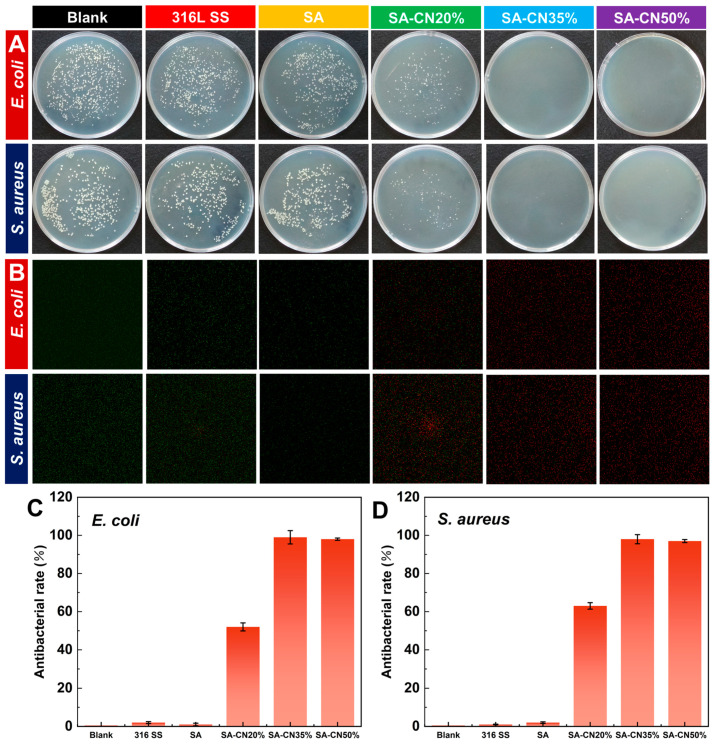
The antibacterial performances of the blank group, 316L SS, SA, SA-CN20%, SA-CN35%, and SA-CN50% against *E. coli* and *S. aureus* are presented in panel (**A**). A confocal laser scanning microscopy (CLSM) diagram is shown in panel (**B**). The antibacterial efficiency against *E. coli* is detailed in panel (**C**), while panel (**D**) illustrates the antibacterial efficiency against *S. aureus*.

SA and SA-CN were formulated into coatings and applied to the surface of 316L stainless steel (316L SS). The antibacterial properties of these coatings were subsequently evaluated. The standard plate counting method was employed to assess the antibacterial efficacy of coatings with varying CN loadings, as illustrated in Figure 4A. Under light conditions, the colony count in the solution that had been in contact with 316L SS was comparable to that of the control group, indicating that the sample exhibited no antibacterial effect. Similarly, the solution in which the SA sample was soaked did not exhibit antibacterial properties under the light conditions. Although the number of colonies soaked in the SA-CN20% solution was significantly reduced, a small number of colonies persisted. This indicates that while SA-CN20% demonstrated antibacterial effects under the light conditions, it did not achieve a 100% antibacterial rate. Notably, when the loading of CN reached 35%, the antibacterial rate against both *E. coli* and *S. aureus* was 100% (Figure 4C,D), which was superior to most sodium alginate hydrogels [47,48,49], nearly consistent with the 50% antibacterial effect observed with SA-CN. This phenomenon occurs because the -OH groups on SA attract the photogenerated holes produced by CN, thereby increasing carrier separation efficiency and enhancing the photocatalytic antibacterial performance [50]. The results indicate that an optimal antibacterial effect is achieved when CN loading reaches 35%. Additionally, even at a CN loading of 50%, while aggregation occurs, a sufficient amount of active oxygen can still be generated, leading to improved bactericidal efficacy.

The durability and long-term antimicrobial properties of the SA-CN35% hydrogels were assessed through immersion experiments, as depicted in Figure S2A,B. The SA-CN35% hydrogel exhibited intact integrity even after one month of immersion in the medium solution containing Escherichia coli and Staphylococcus aureus, indicating excellent durability. Bacterial growth was monitored using absorbance measurements [20]. After one month of immersion, absorbance was measured, as shown in Figure S3A,B. The blank group displayed significantly higher absorbance compared to that of SA-CN35%, suggesting that SA-CN35% effectively inhibited bacterial growth over an extended period, demonstrating outstanding long-term antimicrobial properties.

Live/dead cell staining was employed to further assess the antimicrobial properties of the coating, as illustrated in Figure 4B. Green fluorescence signifies live bacteria, whereas red fluorescence indicates dead bacteria. After 6 h of antibacterial activity under light irradiation, the bacteria in the suspension soaked in SA-CN35% were nearly all dead compared to those in SA, demonstrating an excellent photocatalytic antibacterial performance, which aligns with the colony count results.

### 2.5. Photocatalytic Antibacterial Mechanism

**Figure 5:**
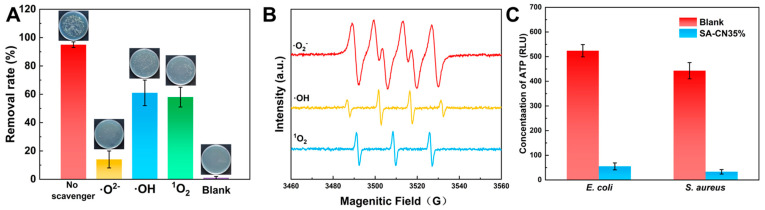
SA-CN35% free radical quenching (**A**); SA-CN35% electron paramagnetic resonance (EPR) of superoxide (·O_2_^−^), hydroxyl radicals (·OH), and singlet oxygen (^1^O_2_) (**B**); C ATP (**C**).

Photocatalysts generate reactive oxygen species (ROS) under light conditions, including superoxide anions (·O_2_^−^), hydroxyl radicals (·OH), and singlet oxygen (^1^O_2_), among others [51]. These reactive oxygen species possess strong oxidizing abilities and can damage the cell membranes and nucleic acids of microorganisms, thus serving an antibacterial function [52]. To investigate the role of various reactive oxygen species in the photocatalytic process of SA-CN35%, 10 mM tert-butanol (TBA), 1,4-benzoquinone (BQ), and L-histidine (L-HIS) were added to quench the hydroxyl radical (·OH), the superoxide radical (·O_2_^−^), and singlet oxygen (^1^O_2_) [53]. As shown in Figure 5A, after 6 h of illumination, the bacteria removal efficiency of SA-CN35% reached an impressive 99.6%. However, upon the addition of TBA, BQ, and L-HIS, the removal rates decreased to 13.2%, 61.8%, and 57.1%, respectively. This indicates that ·O^2-^ plays a crucial role in the photocatalytic antibacterial process of SA-CN35%.

To detect the reactive oxygen species (ROS) generated by SA-CN35%, electron paramagnetic resonance (EPR) measurements were conducted using TEMP and DMPO as spin traps, as illustrated in Figure 5B [54]. The results indicate that superoxide radicals (·O_2_^−^), hydroxyl radicals (·OH), and singlet oxygen (^1^O_2_) produced by SA-CN35% exhibit distinct EPR peaks corresponding to TEMP and DMPO spin adducts (TEMP-^1^O_2_, DMPO-·O_2_^−^, and DMPO-·OH). This confirms that the ROS generated under illumination by SA-CN35% are the primary mechanism driving its photocatalytic antibacterial activity, a conclusion that aligns with the results from quenching experiments and photoelectrochemical analyses.

ATP serves as the primary energy supply molecule in cells and is a crucial marker within the bacterial respiratory chain, providing an indicator for measuring cellular metabolic activity and assessing the antibacterial efficacy of coatings [55]. As illustrated in Figure 5C, it is evident that the ATP level in the soaked 316L SS medium remained largely unchanged compared to that of the control group. In contrast, the ATP content in the soaked sample from the SA-CN35% solution was significantly reduced, measuring only 58 ± 6.8 RLU and 37 ± 5.1 RLU. This finding further substantiates that SA-CN35% possesses superior photocatalytic antibacterial properties.

### 2.6. Corrosion Resistance

**Figure 6:**
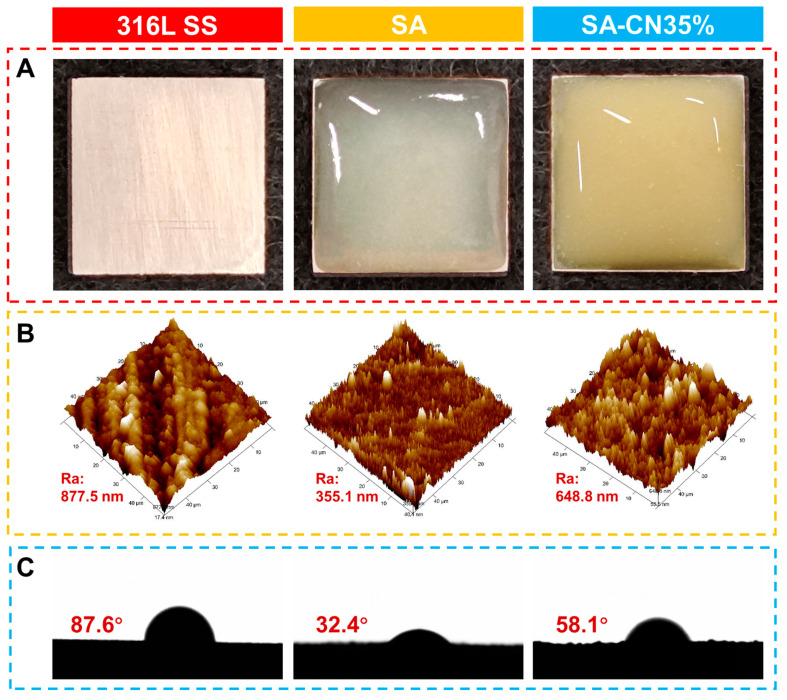
Photographs of 316L, SA, and SA-CN35% coatings (**A**); atomic force microscopy (AFM) images (**B**); and water contact angle measurements (**C**).

To investigate the protective effect of the coating on 316L SS, the coated 316L SS samples were immersed in a bacterial solution for a designated duration, after which the corrosion morphology of the 316L SS surface was examined. Figure 6A illustrates the morphology of the 316L SS samples, along with those coated with SA and sodium alginate loaded with SA-CN35%, respectively. The SA and SA-CN35% coatings exhibit distinct colors; the SA coating is transparent, whereas the SA-CN35% coating, which contains CN, appears light yellow. This color variation can be attributed to the presence of CN, as illustrated in Figures S4 and S5. CN exists as a fine, light-yellow powder. Particle size analysis (Figure S6) indicates that the CN particles are uniformly distributed within the range of 180–260 nm. The even distribution of CN particles in the hydrogel contributes to the observed coating and associated color changes.

The surface morphologies of 316L SS, SA, and SA-CN35% were examined using atomic force microscopy (AFM), as depicted in Figure 6B. The surface roughness of 316L SS was measured at Ra = 877.5 nm. Following the application of the SA coating, a significant reduction in roughness was observed (Ra = 355.1 nm). This improvement can be attributed to the gel coating formed by SA, which possesses excellent sealing properties and results in a smooth, water-based finish on the surface. After the application of SA-CN35%, the surface roughness of the sample increased to Ra = 648.8 nm. This increase can be attributed to the surface height variations resulting from hydrogen bonding between SA and the CN loading. These height differences not only form, but also enhance supply. Additionally, the presence of numerous defects increases the effective surface area, providing a significant number of active sites for photocatalysis, thereby facilitating the progression of photocatalytic reactions [43].

The wettability of the coating significantly influences its corrosion resistance, as illustrated in Figure 6C [56]. The contact angle of the 316L SS surface is measured at 87.6°. Following the application of SA, the contact angle decreased to 32.4°. This reduction is attributed to the high content of hydrophilic oxygen groups, such as -OH and -COOH, present in the SA molecules, which lower the contact angle of the coating and enhance its corrosion resistance. After the addition of CN to SA, the contact angle of SA-CN35% increases to 58.1°. This increase may be attributed to the formation of hydrogen bonds between CN and some of the -OH groups, which reduces the number of exposed hydrophilic groups, and consequently decreases hydrophilicity. Nevertheless, the hydrophilicity of SA-CN35% remains higher than that of 316L SS.

**Figure 7:**
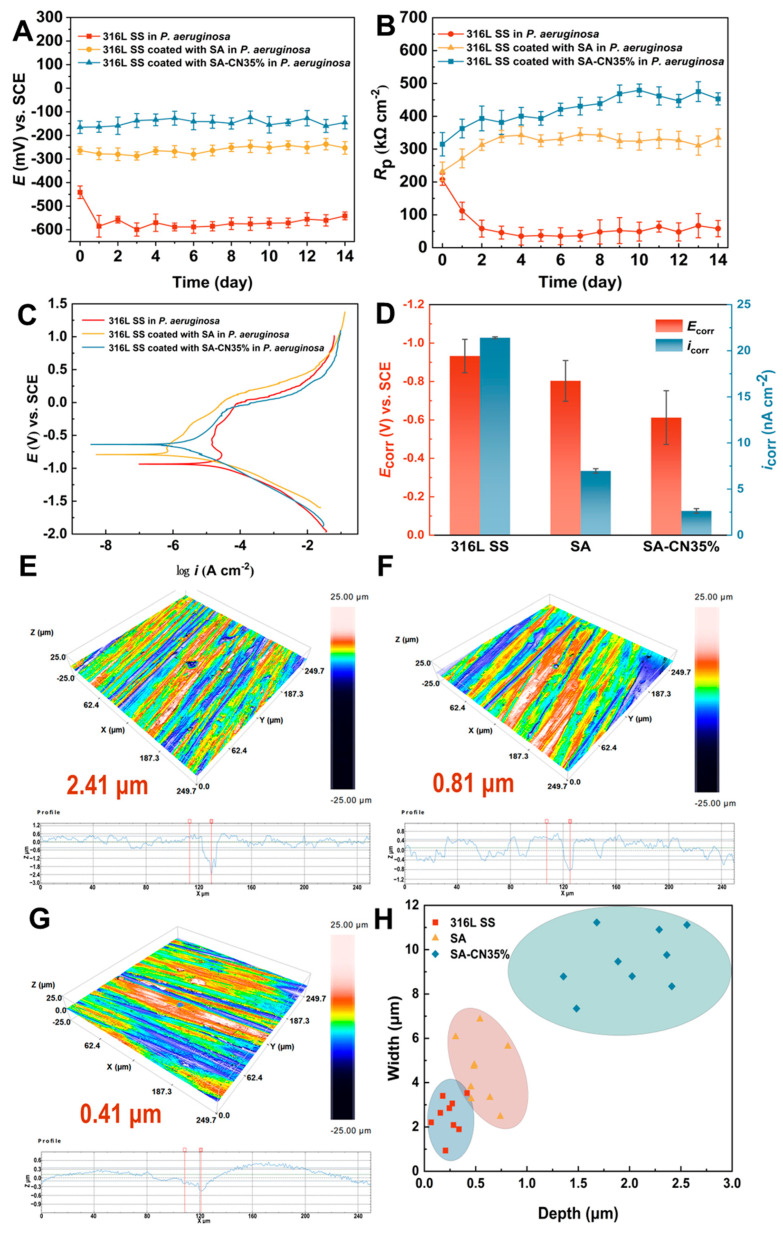
The OCP of the 316L SS, SA, and SA-CN35% coatings (**A**); LPR (**B**); Tafel analysis (**C**); Tafel curve fitting data (**D**); CLSM images of pitting pits (**E**–**G**); and the pitting pit density map (**H**) are presented.

The antibacterial effect of the coating directly influences the antimicrobial corrosion performance of the substrate. *P. aeruginosa* is widely distributed in nature and is the primary bacterial species responsible for the corrosion of metal materials in marine and other environments. Consequently, utilizing *P. aeruginosa* to assess the microbial corrosion resistance of 316L SS, SA, and SA-CN35% holds significant practical importance and serves as a valuable reference [57,58,59]. The open circuit potential (OCP) results (Figure 7A) indicate that SA-CN35% exhibits an *E*_OCP_ correction of −157.6 mV, which was compared to those of 316L SS and SA. The corrosion rate was assessed using linear polarization resistance (LPR) (Figure 7B), and the findings revealed that SA-CN35% demonstrated a higher RP value throughout the 14-day test period in comparison to those of 316L SS and SA, suggesting a lower corrosion rate. Similarly, the potentiodynamic polarization curve (Figure 7C) indicates that the test results for SA-CN35% exhibit a corrected *E*_corr_ and a lower *i*_corr_ (Figure 7D). This suggests that SA-CN35% demonstrates a superior inhibitory effect on the microbial corrosion of 316L SS induced by *P. aeruginosa*.

The pitting morphology of the sample surface was analyzed using confocal laser scanning microscopy (CLSM), as illustrated in Figure 7E–H. After a 14-day culture period in the presence of *P. aeruginosa*, the maximum pitting depth observed on the surface of 316L SS was 2.41 μm, with an average pitting depth of 2.07 ± 0.34 μm. In contrast, the maximum pitting depth of the SA surface was 0.81 μm, with an average depth of 0.7 ± 0.22 μm. The SA-CN35% surface exhibited a maximum pitting depth of only 0.41 μm and an average pitting depth of 0.3 ± 0.25 μm. These results further demonstrate that SA-CN35% possesses superior antimicrobial corrosion properties.

**Figure 8:**
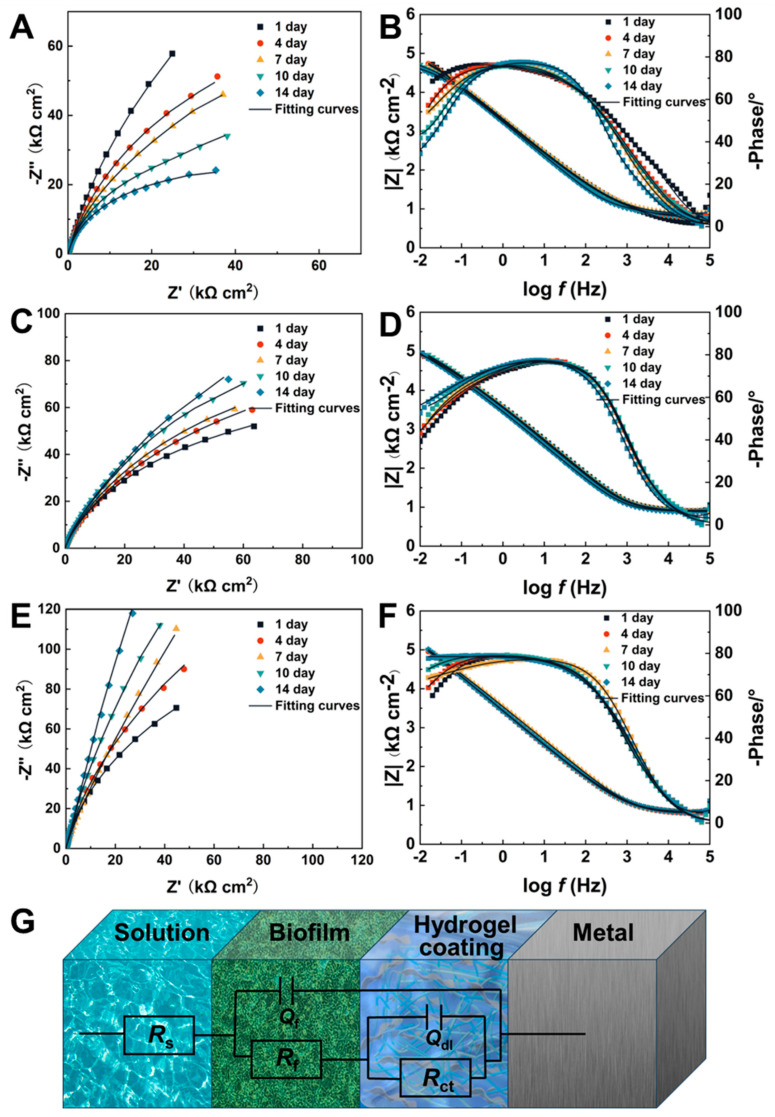
A Nyquist diagram and Bode diagram for 316L (**A**,**B**), SA (**C**,**D**), and SA-CN35% (**E**,**F**) and a fitting circuit diagram (**G**) are presented.

EIS is a technology that has been extensively utilized to assess the corrosion resistance of coatings [60]. Figure 8A–F illustrates Nyquist and Bode plots for the 316L SS (A, B), SA (C, D), and SA-CN35% (E, F) coatings in the Pseudomonas aeruginosa culture medium after 14 days. The size of the capacitive arc in the Nyquist diagram is indicative of the corrosion rate of the sample [61]. Notably, the SA-CN35% coating exhibits the largest capacitive arc radius, signifying superior corrosion resistance.

The impedance modulus (|Z|_0.01Hz_) in the low-frequency Bode plot serves as a crucial indicator of the corrosion resistance of the coating [62]. During the 14-day test, the |Z|_0.01Hz_ of 316L SS exhibited a rapid decline. Although the |Z|_0.01Hz_ of the SA coating increased, it remained at a lower value compared to those of the others. In contrast, the |Z|_0.01Hz_ value of the SA-CN35% coating remained relatively stable and exhibited the highest value.

The circuit diagram presented in Figure 8G was employed to fit the electrochemical data [63]. The EIS data were fitted using the parameters *R*_s_, *R*_f_, *R*_ct_, *C*_b_, and *C*_dl_, which correspond to solution resistance, biofilm resistance, charge transfer resistance, and the electrochemical elements representing biofilm capacitance and the double-layer capacitor, respectively. Table S1 provides a summary of the electrochemical parameters obtained from the fitting of the EIS data for the composite coating.

The EIS data indicate that the corrosion resistance of 316L SS coatings diminishes rapidly in a *P. aeruginosa* medium. The SA coating is unable to prevent bacterial damage over an extended period. In contrast, the SA-CN35% coating demonstrates efficacy in killing and inhibiting *P. aeruginosa*, thereby reducing bacterial damage to the substrate and exhibiting superior corrosion resistance.

## 3. Materials and Methods

### 3.1. Materials and Microorganisms

SA (AR; 120 kDa; G/M ratio, 35/65) was purchased from Aladdin Industrial Corporation (Shanghai, China). Analytically pure calcium carbonate (CaCO_3_), Melamine (98% purity), and acetic acid were purchased from Beijing Tongguang Fine Chemicals Company (Beijing, China). All other reagents were analytical-grade and underwent no further purification throughout the experiment. X80 steel was supplied by the Institute of Metal Research Chinese Academy of Sciences. 316L SS cut into square pieces having dimensions of 10 mm × 10 mm × 5 mm, and subsequently mechanically polished with wet silicon carbide abrasive papers with 240, 400, 600, 800, 1000, and 1200 grits, respectively. Before usage, all coupons were cleaned in distilled water and ethanol for 20 min and sterilized under ultraviolet light for 30 min.

*Pseudomonas aeruginosa* (MCCC 1A 00099) was obtained from the Marine Culture Collection of China (MCCC). *Escherichia coli* (CGMCC 1.8745) and *Staphylococcus aureus* (CGMCC 1.4519) were obtained from the China General Microbiological Culture Collection Center (CGMCC, Beijing, China). 2216 E medium was purchased from Qingdao Hope Biotechnology Co., Ltd. (Qingdao, China) LB medium consisted of the following components (g/L): 5.0 yeast extract, 10.0 peptone, and 10.0 NaCl. PBS buffer contained the following components (g/L): potassium phosphate monobasic (KH_2_PO_4_), 0.27 g; sodium phosphate dibasic (Na_2_HPO_4_), 1.42 g; sodium chloride (NaCl), 8 g; and potassium chloride (KCl), 0.2 g.

### 3.2. Preparation of SA-CN Coating

#### 3.2.1. Preparation of CN

A total of 5 g of melamine was weighed and placed into a 50 mL covered container. Melamine was burnt in a muffle furnace at 550 °C for 2 h, and then allowed to cool naturally to room temperature. This process yielded a yellow solid, which consisted of CN particles.

#### 3.2.2. Preparation of SA-CN Composite Coating

**Figure 9:**
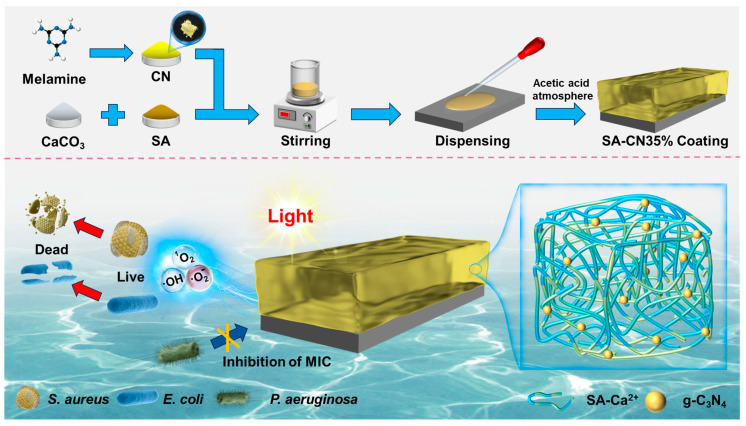
Flow chart of SA-CN35% composite hydrogel preparation.

First, 0.40 g of calcium carbonate was dissolved in 47.8 g of deionized water and stirred for 10 min. Next, 1.75 g of SA was added, and we continued stirring for an additional 10 min. Subsequently, CN was incorporated in varying amounts (0.6125 g) into the mixture. To ensure that carbon nitride was evenly dispersed in the hydrogel, stirring was conducted at 40 °C for 3 h. It was left to sit for 30 min to remove air bubbles. A dropper was used to apply the paddle-like mixture onto the surface of 316L SS. Finally, the samples were placed in an acetic acid atmosphere (100 mL) for 1 h to complete semi-soluble-acidified/sol–gel conversion. During the conversion process, CN was anchored in sol to prevent uneven precipitation, and a uniform SA-CN gel coating was obtained after conversion as shown in Figure 9. The resulting samples were designated as SA, SA-CN20%, SA-CN35%, and SA-CN50%.

### 3.3. Characterization

The surface morphology and structure of the samples were observed using a field emission scanning electron microscope (SEM, Zeiss Gemini SEM 500, Oberkochen, Germany). Energy dispersive spectroscopy (EDS, INCAx-Sight 6427, Tokyo, Japan) was employed to analyze the elemental distribution within the samples. The chemical structure of the samples was characterized using Fourier-transform infrared spectroscopy (FT-IR, SHIMADZU IRPrestige-21, Kenilworth, NJ, USA) over a scanning range of 400–4000 cm⁻^1^. The crystal structure was characterized using X-ray diffraction (XRD, Ultima IV, Cu Kα, Waltham, MA, USA) within a 2θ range of 5–80° at a step size of 0.02°. Morphological images of the samples were obtained through atomic force microscopy (AFM, Bruker Multimode 8, Billerica, MA, USA) in contact mode. The contact angle of the samples was measured using an SL200B contact angle meter (CA, Kono Industries, Ltd., Seattle, WA, USA). The photoluminescence spectrum was recorded using a fluorescence spectrometer (PL, Hitachi F-4600, Tokyo, Japan) to assess the recombination efficiency of the photogenerated carriers. Ultraviolet-visible diffuse reflectance spectroscopy (UV-vis DRS, U-3900 H, Kyoto, Japan) was utilized to evaluate the absorption edge of the sample, and the band gap energy was calculated using the Kubelka–Munk function (1) [64].

(1) $$\alpha hv=A{\left(hv-E_{g}\right)}^{\frac{1}{2}}$$

### 3.4. Mechanical Performance Testing

The tensile properties of the hydrogels with and without CN were measured using an XT Plus C texture analyzer (Stable Micro Systems, Godalmin, UK) [65]. All the composite hydrogels were cut into dumbbell shapes, with a length of 50 mm and a width of 10 mm. The testing speed was set at 1 mm/s, and the initial distance was 35 mm. To ensure the accuracy and reliability of the experimental results, the relevant experiments were repeated three times.

The samples were freeze-dried at −60 °C until a constant weight was achieved, designated as *M*_0_. Four species were then immersed in 20 mL of deionized water for 150 min. After this period, the samples were removed, and any residual water was wiped off. The weight of the samples was recorded as *M*_1_. The swelling rate was calculated using Equation (2) [66].

(2) $$Swelling\;rate=\frac{M_{0}-M_{1}}{M_{0}}\times 100\%$$

### 3.5. Antibacterial Performance Testing

The plate counting method was employed to evaluate the antibacterial activity of the samples under both light and dark conditions. The concentration of the bacterial solution was standardized to 1 × 10^7^ CFU/mL. The samples were introduced into glass test tubes containing the bacterial solution and were maintained in darkness or light for 6 h, respectively. The control group consisted of a bacterial solution at the same concentration without the addition of hydrogel. Subsequently, 50 μL of the bacterial solution was spread onto a solid agar medium. After a 24 h incubation period, the colonies on the solid medium were counted, and the antibacterial rate was calculated using Equation (3).

(3) $$Antibacterial\;rate=\frac{{CFU}_{0}-CFU}{{CFU}_{0}}\times 100\%$$

Cell viability staining was also employed to further characterize the antibacterial properties of the samples. The sample surface was stained using a bacterial viability kit (LIVE/DEAD^®^ BacLightTM, Invi Biotrogen, Carlsbad, CA, USA) [67]. After 15 min incubation in a dark environment, live cells (green) and dead cells (red) were observed using a confocal laser scanning microscope (CLSM, LSM 900, Zeiss, Oberkochen, Germany).

The ATP concentration was measured using an ATP fluorescence detector (UPF-10ATP, Up General, Beijing, China). After 6 h of antibacterial treatment, 2 mL of the bacterial suspension was withdrawn. The cotton tip of an adenosine triphosphate (ATP) sampling swab was immersed into the bacterial liquid to initiate the measurement, and this process was repeated three times.

To ascertain the precision and dependability of the experimental outcomes, each pertinent antibacterial experiment was meticulously replicated three times.

### 3.6. Photocatalytic Performance Testing

The photocatalytic antibacterial mechanism was elucidated through free radical quenching experiments. Tert-butyl alcohol (TBA, 1 mM), 1,4-benzoquinone (BQ, 1 mM), and L-histidine (L-HIS, 0.5 mM) were introduced to the bacterial suspension containing SA-CN35% hydrogel to capture hydroxyl radicals (·OH), superoxide radicals (·O_2_^−^), and singlet oxygen (^1^O_2_). The experimental procedure mirrored that of the photo antibacterial experiment, with the exception that the quenchers were added in advance.

The species ·O_2_^−^, ^1^O_2_, and ·OH were detected using an electron spin resonance spectrometer (ESR, Billerica, USA).

### 3.7. Optoelectronic Performance Testing

The TPC and EIS of the sample were measured using a Princeton electrochemical workstation (Reference 600+, Gamry Instruments, Philadelphia, PA, USA). The sample served as the working electrode, while a platinum wire was utilized as the counter electrode, and a saturated calomel electrode functioned as the reference electrode. Measurements were conducted using a standard three-electrode system, with a 300 W Xe lamp employed as the light source.

### 3.8. Corrosion Resistance Test

The same electrochemical workstation used in Section 3.7 was employed to assess the corrosion behavior of the samples. The test was conducted using a three-electrode system containing 250 mL of 2216E culture medium, with a concentration of 1 × 10^7^ CFU/mL of *P. aeruginosa* serving as the experimental medium. The initial pH of the medium was adjusted to 8.0. LPR, EIS, and potentiodynamic polarization curves of the samples in culture were measured utilizing a potential scanning speed of 0.125 mV/s and a scanning range of ±10 mV (vs. OCP). Linear polarization resistance was determined, and the EIS data were collected over a frequency range of 10^5^ to 10^−2^ Hz, with an amplitude of 5 mV. And the soaking time for the OCP, LPR, and EIS experiments was 14 days. After 14 days, polarization curves were recorded at a scan rate of 0.17 mV/s within a potential range from −1.5 to +1.5 V (vs. *E*_OCP_). The tests were conducted under 35 °C.

After soaking for 14 days, the corrosion products, the biofilm, and the composite hydrogel coating were removed, and the pit depth was measured using confocal laser scanning microscopy (CLSM) (LSM 710, Zeiss, Jena, Germany). For pit testing, three samples were immersed simultaneously in the same Erlenmeyer flask.

## 4. Conclusions

This study successfully demonstrates the potential application of SA and CN eco-friendly composite hydrogel coatings in the fields of antibacterial and anticorrosion research.

(1) The electrostatic interactions between SA and Ca^2+^, as well as hydrogen bonding between SA and CN, indicate that CN was effectively anchored within the SA matrix. This arrangement significantly enhances the dispersion of CN and imparts excellent antibacterial properties and mechanical strength to the hydrogel coating.

(2) The mechanisms underlying the effects of CN loading on the mechanical and photocatalytic properties of the coating were also investigated. At a CN content of 35%, the hydrogel coating exhibited a tensile strength of up to 120 MPa and a photocatalytic antibacterial rate of 99.87%, while maintaining high antibacterial efficacy after multiple uses.

(3) Mechanistic analysis revealed the critical role of SA in facilitating the transfer of photogenerated carriers. These findings provide the scientific basis for the development of new, efficient antibacterial and anticorrosive materials.

(4) The coating demonstrates a significant inhibitory effect on the microbial corrosion of stainless steel caused by *P. aeruginosa*, indicating its promising prospects for practical applications.

## Data Availability

Data are contained within this article and the Supplementary Materials.

## Supplementary Materials

The following supporting information can be downloaded at: https://www.mdpi.com/article/10.3390/molecules29174192/s1, Figure S1. The EDS proportion image of SA-CN35%. Figure S2. Under lighting conditions, photograph of the hydrogel after soaking in *E. coli* medium for 1 month (A), *S. aureus* (B). Figure S3. Under light conditions, the absorbance of the hydrogel in the *E. coli* culture medium after immersion for 1 month. (A), *S. aureus* (B). Figure S4. Photographs of CN. Figure S5. SEM image of CN. Figure S6. Analysis of Carbon Nitride Particle Size. Table S1. Electrochemical model impedance parameters of coupons incubated in *P. aeruginosa* medium.

## References

[1] Jing J., Guo J., Li B., Jia S., Ren Y. (2021). Relationship between Microstructure and Corrosion Behavior of High-Grade Pipeline Steel in a Low-Temperature Environment. J. Iron Steel Res. Int..

[2] Wang J., Du M., Shan X. (2023). Effect of Marine Streptomyces on Corrosion Behavior of X65 Steel in Simulated Offshore Oilfield Produced Water System. J Mater Res Technol.

[3] Bhandari J., Khan F., Abbassi R., Garaniya V., Ojeda R. (2015). Modelling of Pitting Corrosion in Marine and Offshore Steel Structures—A Technical Review. J. Loss Prev. Process. Ind..

[4] Feng J., Feng Q., Xin J., Liang Q., Li X., Chen K., Teng J., Wang S., Feng L., Liu J. (2023). Fabrication of Durable Self-Cleaning Photocatalytic Coating with Long-Term Effective Natural Light Photocatalytic Degradation Performance. Chemosphere.

[5] Kang L., Peng H., Yang M., Hu K., Lin Y., Zhu Y., Chen H., Zhao J., Han S., Wang Y. (2024). Dual-Functional Acridine-Based Coatings with Anti-Bacterial Adhesion and Durable Photocatalytic Antibacterial. Prog. Org. Coat..

[6] Fattah-alhosseini A., Chaharmahali R., Alizad S., Babaei K., Stojadinović S. (2024). A Review on the Revealed Improved Photocatalytic Activity of PEO Coatings Applied on Al Alloys. Nano-Struct. Nano-Objects.

[7] Cao Q., Kumru B., Antonietti M., Schmidt B.V.K.J. (2020). Graphitic Carbon Nitride and Polymers: A Mutual Combination for Advanced Properties. Mater. Horiz..

[8] Yuan S., Dai L., Xie M., Liu J., Peng H. (2024). Modification Optimization and Application of Graphitic Carbon Nitride in Photocatalysis: Current Progress and Future Prospects. Chem. Eng. Sci..

[9] Hao J., Yan S., Yuan H., Du C., Tan Y. (2024). High-Strength Alginate Fibers Wet-Spun from Pre-Crosslinked Sodium Alginate Solutions. Carbohydr. Polym..

[10] Xie F. (2024). Alginate-Based Nanocomposites for Food Preservation: Recent Progress Showcasing Heightened Material Properties and Functionalities. Adv. Nanocompos..

[11] Mandal S., Sarkar A., Mukherjee P., Das S., Banerjee D., Ganguly S., Kargupta K. (2024). Organic Alginate Encapsulated rGO-CdS Millispheres for Remarkable Photocatalytic Solar Hydrogen Production. Int. J. Hydrogen Energy.

[12] Khan S.B., Ahmad S., Kamal T., Asiri A.M., Bakhsh E.M. (2020). Metal Nanoparticles Decorated Sodium Alginate-carbon Nitride Composite Beads as Effective Catalyst for the Reduction of Organic Pollutants. Int. J. Biol. Macromol..

[13] Zhan X., Zhang Z., Lin J., Xu J., Wang X., Hong B., Xia Y., Zeng Y. (2024). Surface Atom Rearrangement Enabling Graphitic Carbon Nitride/Sodium Alginate Gel Monolith for Ultrafast Completely Photodegrading Ciprofloxacin under Visible Light. Chem. Eng. J..

[14] Kong D., Yang B., Yuan H., Du C., Tan Y. (2024). 3D Printing of Glycerol-Mediated Alginate Hydrogels with High Strength and Stiffness. J. Mater. Sci. Technol..

[15] Cao X., Ma L., Tan Y., Tong Q., Liu D., Yi Z., Li X. (2024). Soft yet Mechanically Robust Injectable Alginate Hydrogels with Processing Versatility Based on Alginate/Hydroxyapatite Hybridization. Int. J. Biol. Macromol..

[16] Wang J., Zhang Z., Zhu J., Tian M., Zheng S., Wang F., Wang X., Wang L. (2020). Ion Sieving by a Two-Dimensional Ti_3_C_2_Tx Alginate Lamellar Membrane with Stable Interlayer Spacing. Nat. Commun..

[17] Bhat S.D., Aminabhavi D.T.M. (2007). Pervaporation Separation Using Sodium Alginate and Its Modified Membranes—A Review. Sep. Purif. Rev..

[18] Ye C., Gong Q.-M., Lu F.-P., Liang J. (2008). Preparation of Carbon Nanotubes/Phenolic-Resin-Derived Activated Carbon Spheres for the Removal of Middle Molecular Weight Toxins. Sep. Purif. Technol..

[19] Jiang H., Yang Y., Lin Z., Zhao B., Wang J., Xie J., Zhang A. (2020). Preparation of a Novel Bio-Adsorbent of Sodium Alginate Grafted Polyacrylamide/Graphene Oxide Hydrogel for the Adsorption of Heavy Metal Ion. Sci. Total Environ..

[20] Zhang M., Sun T., Wang X.-Y., Xue B. (2024). Photo-Crosslinked Composite Hydrogels with Silver-Deposited Polymeric Carbon Nitride for Boosting Antibacterial Activity. Colloid. Surface A.

[21] Zhang J., Yu G., Yang C., Li S. (2024). Recent Progress on S-Scheme Heterojunction Strategy Enabling Polymer Carbon Nitrides CN and CN Enhanced Photocatalysis in Energy Conversion and Environmental Remediation_3435_. Curr. Opin. Chem. Eng..

[22] Huang Q., Yu J., Cao S., Cui C., Cheng B. (2015). Efficient Photocatalytic Reduction of CO_2_ by Amine-Functionalized g-C_3_N_4_. Appl. Surf. Sci..

[23] Li Y., Xu H., Ouyang S., Lu D., Wang X., Wang D., Ye J. (2016). In Situ Surface Alkalinized G-C_3_N_4_ toward Enhancement of Photocatalytic H_2_ Evolution under Visible-Light Irradiation. J. Mater. Chem. A.

[24] You Z., Wang C., Hu P., Zhang W., Li Q., Zheng Y. (2024). Construction of Dual Driving Force in Carbon Nitride for Highly Efficient Hydrogen Evolution: Simultaneously Manipulating Carriers Transport in Intra- and Interlayer. J. Colloid. Interface Sci..

[25] Tang S., Yang J., Lin L., Peng K., Chen Y., Jin S., Yao W. (2020). Construction of Physically Crosslinked Chitosan/Sodium Alginate/Calcium Ion Double-Network Hydrogel and Its Application to Heavy Metal Ions Removal. Chem. Eng. J..

[26] Hao D., Huang Q., Wei W., Bai X., Ni B.-J. (2021). A Reusable, Separation-Free and Biodegradable Calcium Alginate/g-CN Microsphere for Sustainable Photocatalytic Wastewater Treatment_34_. J. Clean. Prod..

[27] Fan J., Shi Z., Lian M., Li H., Yin J. (2013). Mechanically Strong Graphene Oxide/Sodium Alginate/Polyacrylamide Nanocomposite Hydrogel with Improved Dye Adsorption Capacity. J. Mater. Chem. A.

[28] Zheng Y., Lin L., Ye X., Guo F., Wang X. (2014). Helical Graphitic Carbon Nitrides with Photocatalytic and Optical Activities. Angew. Chem. Int. Ed. Engl..

[29] Dalponte I., de Sousa B.C., Mathias A.L., Jorge R.M.M. (2019). Formulation and Optimization of a Novel TiO_2_/Calcium Alginate Floating Photocatalyst. Int. J. Biol. Macromol..

[30] Nawaz M., Khan A.A., Hussain A., Jang J., Jung H.-Y., Lee D.S. (2020). Reduced Graphene oxide-TiO_2_/Sodium Alginate 3-Dimensional Structure Aerogel for Enhanced Photocatalytic Degradation of Ibuprofen and Sulfamethoxazole. Chemosphere.

[31] Nawaz M., Moztahida M., Kim J., Shahzad A., Jang J., Miran W., Lee D.S. (2018). Photodegradation of Microcystin-LR Using Graphene-TiO_2_/Sodium Alginate Aerogels. Carbohyd Polym..

[32] Fang X., Feng C., Li T., Wang Y., Zhu S., Ren H., Huang H. (2023). G-C_3_N_4_/Polyvinyl Alcohol-Sodium Alginate Aerogel for Removal of Typical Heterocyclic Drugs from Water. Environ. Pollut..

[33] Balakrishnan A., Chinthala M., Polagani R.K. (2024). 3D Kaolinite/g-C_3_N_4_-Alginate Beads as an Affordable and Sustainable Photocatalyst for Wastewater Remediation. Carbohydr. Polym..

[34] Guerrero J.D., Arias E.R., Gutierrez L.B. (2024). Enhancing Copper and Lead Adsorption in Water by In-Situ Generation of Calcium Carbonate on Alginate/Chitosan Biocomposite Surfaces. Int. J. Biol. Macromol..

[35] Divakar S., Sampatkumar H.G., Naik S.S., Malladi S., Padaki M., Patil S.A., Balakrishna R. (2023). Graphitic Carbon Nitride Enriched Phytochemicals-Based Integrated Membranes for Perilous Chromium (VI) Ion Removal. Sep. Purif. Technol..

[36] Zhang Y., Wu G., Feng F., Gao C., Jin X., Zhang X., Xing W. (2024). Synergetic Effects of In-Plane and Interlayer Dual Regulation on Sandworm-like Graphitic Carbon Nitride for High-Efficiency Photocatalytic Performance. Opt. Mater..

[37] Valiyathur M.F., Kottur A.B., Sakvai M.S. (2024). Alginate Reinforced Composite of CuO-gCN: Synthesis, Characterization and Photocatalytic Activity34. Mater Lett.

[38] Falletta E., Longhi M., Di Michele A., Boffito D.C., Bianchi C.L. (2022). Floatable Graphitic Carbon Nitride/Alginate Beads for the Photodegradation of Organic Pollutants under Solar Light Irradiation. J. Clean. Prod..

[39] Li P., Xie S., Wang G., Qiu L., Liu Y., Liu J. (2024). Surface Electric Dipole and Coordinate Microenvironment Mediated Charge Separation and Transfer for Patterned Carbon Nitride Photocatalysis. Appl. Surf. Sci..

[40] Sun S., Li C., Sun Z., Wang J., Wang X., Ding H. (2021). In-Situ Design of Efficient Hydroxylated SiO_2_/g-C_3_N_4_ Composite Photocatalyst: Synergistic Effect of Compounding and Surface Hydroxylation. Chem. Eng. J..

[41] Xia L., Huang H., Fan Z., Hu D., Zhang D., Khan A.S., Usman M., Pan L. (2019). Hierarchical Macro-/Meso-/Microporous Oxygen-Doped Carbon Derived from Sodium Alginate: A Cost-Effective Biomass Material for Binder-Free Supercapacitors. Mater. Design.

[42] Zhao R., Ke Y., Maschmeyer T., Li X. (2022). O_2_-Promoted Photodoping for Enhanced Photocurrent on Polymeric Carbon Nitride. Mater. Today Sustain..

[43] Zhao X., Wang X., Lv Y., Zhao W., Dong Y., Wang L. (2023). Sodium Alginate Intercalated 2D G-CN Membrane: Efficient Dye Removal and Photocatalytic Self-Cleaning_34_. Sep. Purif. Technol..

[44] Saeidi Tabar F., Pourmadadi M., Yazdian F., Rashedi H., Rahdar A., Fathi-karkan S., Romanholo Ferreira L.F. (2024). Ultrasensitive Aptamer-Based Electrochemical Nanobiosensor in Diagnosis of Prostate Cancer Using 2D:2D Reduced Graphene Oxide/Graphitic Carbon Nitride Decorated with Au Nanoparticles. Eur. J. Med. Chem. Rep..

[45] Peng M., Zhang Q., Zhang M., Yang Z., Wan Y., Deng X., Luo H. (2023). Dealloying and Polydopamine/Silver Coating on NiTi Alloy for Improved Antibacterial Activity. Mater. Chem. Phys..

[46] Li Z., Liu L., Zheng H., Meng F., Wang F. (2022). Thermoresponsive PNIPAm on Anti-Corrosion Antibacterial Coating for Controlled Ag Ions Release. Compos. Commun..

[47] El-Sayed N.S., Hashem A.H., Khattab T.A., Kamel S. (2023). New Antibacterial Hydrogels Based on Sodium Alginate. Int. J. Biol. Macromol..

[48] Zhang J., Zhang S., Liu C., Lu Z., Li M., Hurren C., Wang D. (2024). Photopolymerized Multifunctional Sodium Alginate-Based Hydrogel for Antibacterial and Coagulation Dressings. Int. J. Biol. Macromol..

[49] Feyissa Z., Edossa G.D., Gupta N.K., Negera D. (2023). Development of Double Crosslinked Sodium Alginate/Chitosan Based Hydrogels for Controlled Release of Metronidazole and Its Antibacterial Activity. Heliyon.

[50] Vigneshwaran S., Preethi J., Meenakshi S. (2019). Removal of Chlorpyrifos, an Insecticide Using Metal Free Heterogeneous Graphitic Carbon Nitride (g-CN) Incorporated Chitosan as Catalyst: Photocatalytic and Adsorption Studies_34_. Int. J. Biol. Macromol..

[51] Shen H., Durkin D.P., Aiello A., Diba T., Lafleur J., Zara J.M., Shen Y., Shuai D. (2021). Photocatalytic Graphitic Carbon Nitride-Chitosan Composites for Pathogenic Biofilm Control under Visible Light Irradiation. J. Hazard..

[52] Latif M.J., Ali S., Jamil S., Bibi S., Jafar T., Rasheed A., Noreen S., Bashir A., Rauf Khan S. (2024). Comparative Catalytic Reduction and Degradation with Biodegradable Sodium Alginate Based Nanocomposite: Zinc Oxide/N-Doped Carbon Nitride/Sodium Alginate. Int. J. Biol. Macromol..

[53] Chitra V.P., Vasantharani P., Sivakumar G., Arif Dar M., Majid S.R., Rezaul Karim M., Arularasan P., Guganathan L. (2024). Structural, Morphological and Optical Properties of CuAl_2_O_4_ Nanoparticles and Their Applications for Photocatalytic and Antibacterial Activities. Inorg. Chem. Commun..

[54] Chen Y., Wang X., Zeng Z., Lv M., Wang K., Wang H., Tang X. (2024). Towards Molecular Understanding of Surface and Interface Catalytic Engineering in TiO_2_/TiOF_2_ Nanosheets Photocatalytic Antibacterial under Visible Light Irradiation. J. Hazard..

[55] Zhang M., Liu S., Gao X., Jiang X., Zhang E., Fan H., Zhu S. (2024). Highly Flexible Carbon Nitride-Polyethylene Glycol-Cellulose Acetate Film with Photocatalytic Antibacterial Activity for Fruit Preservation. Int. J. Biol. Macromol..

[56] Wang B.-B., Quan Y.-H., Xu Z.-M., Zhao Q. (2020). Preparation of Highly Effective Antibacterial Coating with Polydopamine/Chitosan/Silver Nanoparticles via Simple Immersion. Prog. Org. Coat..

[57] Liu J., Jia R., Zhou E., Zhao Y., Dou W., Xu D., Yang K., Gu T. (2018). Antimicrobial Cu-Bearing 2205 Duplex Stainless Steel against MIC by Nitrate Reducing *Pseudomonas aeruginosa* Biofilm. Int. Biodeterior..

[58] Liu D., Yang H., Li J., Li J., Dong Y., Yang C., Jin Y., Yassir L., Li Z., Hernandez D. (2021). Electron Transfer Mediator PCN Secreted by Aerobic Marine *Pseudomonas aeruginosa* Accelerates Microbiologically Influenced Corrosion of TC4 Titanium Alloy. J. Mater. Sci. Technol..

[59] Zhou X., Zhou Z., Wu T., Li C., Li Z. (2021). Effects of Non-Viable Microbial Film on Corrosion of Pipeline Steel in Soil Environment. Corr. Comm..

[60] Liu D., Hu Z., Li M., Han B., Liang Y., Dilawer Hayat M., Sun Y., Jin D., Li J., Wang B. (2024). Synergistic Effect on Corrosion Behavior of X80 Steel Influenced by *Pseudomonas aeruginosa* and *Acetobacter aceti*. Sep. Purif. Technol..

[61] Li M., Hu Z., Liu D., Liang Y., Liu S., Wang B., Niu C., Xu D., Li J., Han B. (2024). Efficient Antibacterial and Microbial Corrosion Resistant Photocatalytic Coating: Enhancing Performance with S-Type Heterojunction and Cu Synergy. Chem. Eng. J..

[62] Li M., Zhou J., Li Y., Zhu G., Hu Z., Liu S., Han B., Zhao H., Liang Y., Liu D. (2024). Enhanced Antibacterial and Corrosion Resistance of Copper-Containing 2205 Duplex Stainless Steel against the Corrosive Bacterium *Shewanella algae*. Bioelectrochemistry.

[63] Liu D., Liang Y., Wei H., Liu P., Jin D., Yassir L., Han B., Li J., Xu D. (2024). Enhanced Corrosion of 2205 Duplex Stainless Steel by *Acetobacter aceti* through Synergistic Electron Transfer and Organic Acids Acceleration. Bioelectrochemistry.

[64] Jubu P.R., Danladi E., Ndeze U.I., Adedokun O., Landi S., Haider A.J., Adepoju A.T., Yusof Y., Obaseki O.S., Yam F.K. (2024). Comment about the Use of Unconventional Tauc Plots for Bandgap Energy Determination of Semiconductors Using UV–Vis Spectroscopy. Results Opt..

[65] Wan J., Wang R., Liu Z., Zhang S., Hao J., Mao J., Li H., Chao D., Zhang L., Zhang C. (2024). Hydrated Eutectic Electrolyte Induced Bilayer Interphase for High-Performance Aqueous Zn-Ion Batteries with 100 °C Wide-Temperature Range. Adv. Mater..

[66] Zhao C.-X., Liu J.-N., Li B.-Q., Ren D., Chen X., Yu J., Zhang Q. (2020). Multiscale Construction of Bifunctional Electrocatalysts for Long-Lifespan Rechargeable Zinc–Air Batteries. Adv. Funct..

[67] Wang Q., Wang B., Zhou X., Tan Z., Zhang M., Luo J., Wang Y., Wu T. (2024). Effects of Carbon Source Starvation and *Riboflavin* Addition on Selective Corrosion of Welded Joint by *Desulfovibrio vulgaris*. Corros. Sci..

