# Supplementary material for: Antibacterial and Anticorrosive Hydrogel Coating Based on Complementary Functions of Sodium Alginate and g-C3N4
Source: Molecules. 2024 Sep 4;29(17):4192. doi: 10.3390/molecules29174192 (PMC11396995; doi:10.3390/molecules29174192)
Supplement: Supplementary file 1 [file molecules-29-04192-s001.zip › molecules-3175458-supplementary.pdf]

Supporting Information

Antibacterial and anticorrosive hydrogel coating based on complementary functions of sodium alginate and g-C_3N_4

Zishuai Hu [1], Baochen Han[1,*], Jianhui Li[1], Dan Liu [1,*] and Jian Qi [2,*]

[1]Hebei Short Process Steelmaking Technology Innovation Center, School of Materials Science and Engineering, Hebei University of Science and Technology, Shijiazhuang 050018, China

[2]State Key Laboratory of Biochemical Engineering, Institute of Process Engineering, Chinese Academy of Sciences, Beijing 100049, China

*Correspondence: hbchebust@163.com (B. Han); danliu217@163.com (D. L.); jqi@ipe.ac.cn (J. Q.)

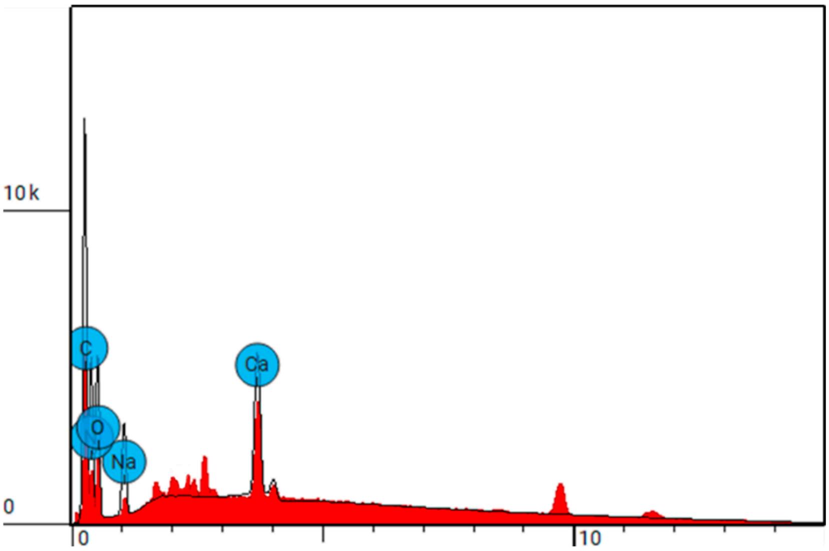

Figure S1. The EDS proportion image of SA-CN35%

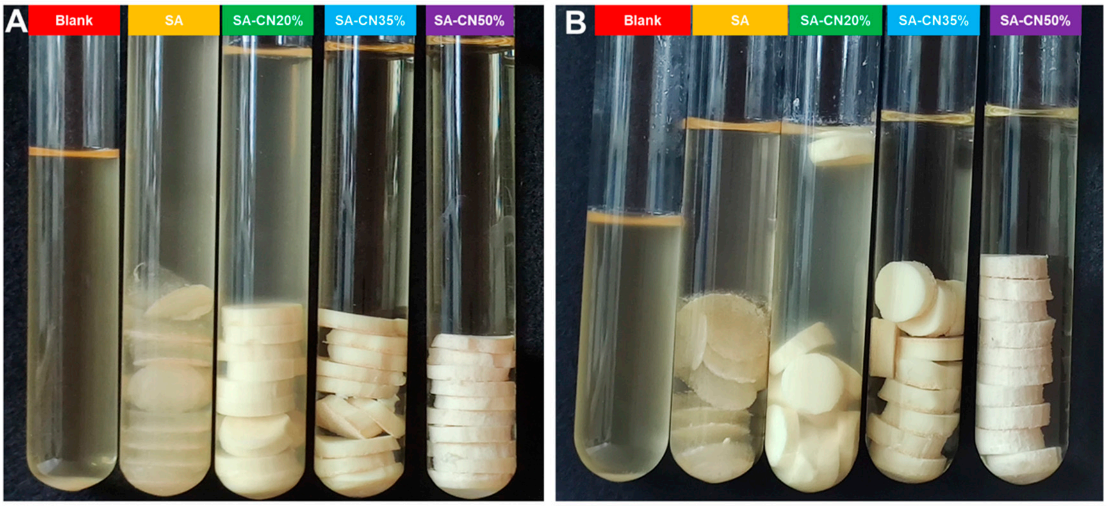

Figure S2. Under lighting conditions, photograph of the hydrogel after soaking in *E. coli* medium for 1 month (A), *S. aureus* (B).

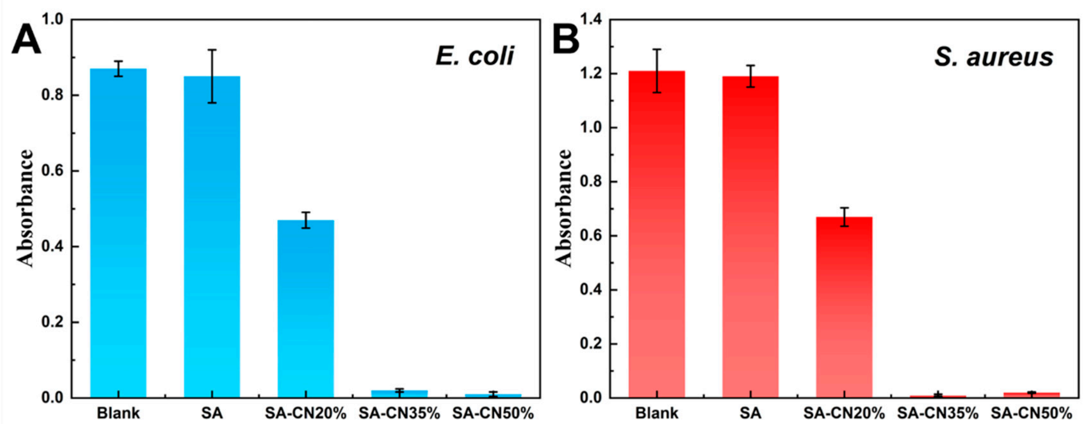

Figure S3. Under light conditions, the absorbance of the hydrogel in the *E. coli* culture medium after immersion for 1 month. (A), *S. aureus* (B).

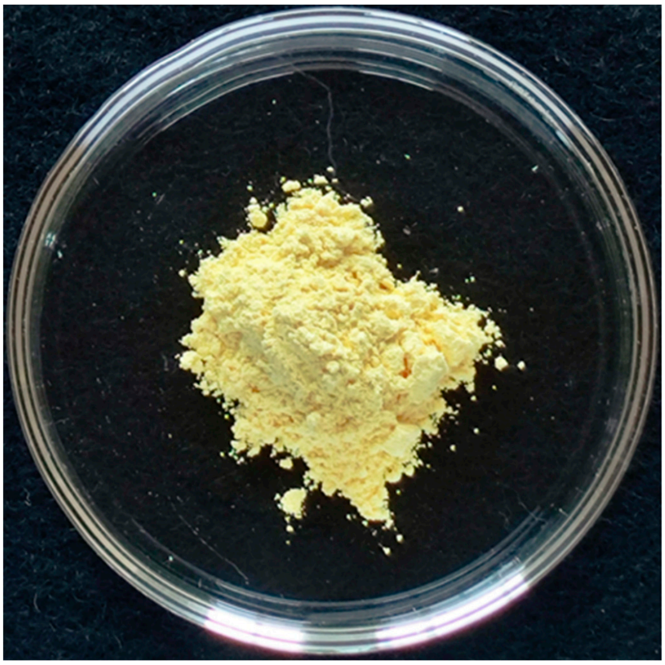

Figure S4. Photographs of CN

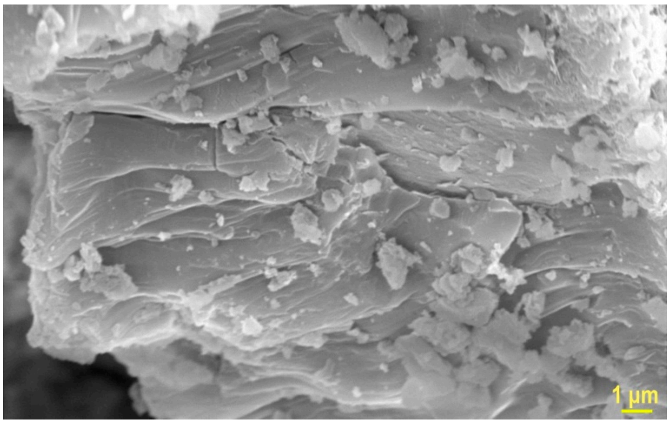

Figure S5. SEM image of CN

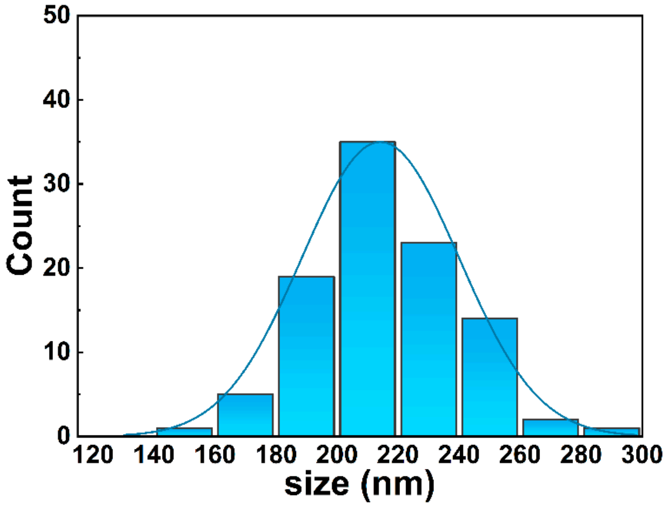

Figure S6. Analysis of Carbon Nitride Particle Size

Table S1. Electrochemical model impedance parameters of coupons incubated in *P. aeruginosa* medium.

Time/d	R_S/ ($\Omega\cdot cm^2$)	C_b/ 10^{-5} ($\mu F\cdot cm^{-2}$)	R_f/ ($\Omega\cdot cm^2$)	C_{dl}/10^{-3} ($\mu F\cdot cm^{-2}$)	R_{ct}/ ($k\Omega\cdot cm^2$)
316L SS in *P. aeruginosa* medium					
1	4.49	1.01	21.9	2.01	39.9
4	6.07	1.83	8.89	0.78	37.5
7	7.73	7.19	4.60	0.37	15.3
10	8.17	7.64	4.26	0.11	4.28
14	8.31	7.91	3.17	0.03	0.04
316L SS coated with SA in *P. aeruginosa* medium					
1	8.37	7.91	6.18	0.06	54.2
4	8.22	2.20	6.24	0.08	65.7
7	7.89	1.41	6.47	0.09	69.2
10	7.68	1.32	6.82	0.15	96.1
14	7.32	1.29	7.62	0.72	97.2
316L SS coated with SA-CN35% in *P. aeruginosa* medium					
1	11.81	1.42	3.06	1.42	71.33
4	11.77	1.32	3.97	1.51	83.14
7	10.97	1.31	4.03	1.88	107.71
10	10.68	1.21	4.62	1.97	129.51
14	10.52	1.17	6.05	2.27	142.73
